# A Longitudinal Study Examining Adherence to Guidelines in Diabetes Care According to Different Definitions of Adequacy and Timeliness

**DOI:** 10.1371/journal.pone.0024278

**Published:** 2011-09-08

**Authors:** Grigory Sidorenkov, Flora M. Haaijer-Ruskamp, Dick de Zeeuw, Petra Denig

**Affiliations:** 1 Department of Clinical Pharmacology, University Medical Center Groningen, University of Groningen, Groningen, The Netherlands; 2 Research Institute SHARE of the Graduate School of Medical Sciences, University Medical Center Groningen, University of Groningen, Groningen, The Netherlands; Yale University School of Medicine, United States of America

## Abstract

**Background:**

Performance indicators assessing quality of diabetes care often look at single processes, e.g. whether an HbA1c test was conducted. Adequate care, however, consists of consecutive processes which should be taken in time (clinical pathways). We assessed quality of diabetes care by looking at single processes versus clinical pathways. In addition, we evaluated the impact of time period definitions on this quality assessment.

**Methodology:**

We conducted a cohort study in 2007–2008 using the GIANTT (Groningen Initiative to Analyse type 2 diabetes Treatment) database. Proportions of patients adequately managed for HbA1c, systolic blood pressure (SBP), LDL-cholesterol (LDL-C), and albumin/creatinin ratio (ACR) were calculated for the pathway of (1) risk factor level testing, (2) treatment intensification when indicated, (3) response to treatment evaluation. Strict and wide time periods for each step were defined. Proportions of patients adequately managed regarding the overall pathway and single steps, using strict or wide time periods were compared using odds ratios (OR) with 95% confidence intervals.

**Findings:**

Of 11176 patients diagnosed with type 2 diabetes, 9439 with complete follow-up were included. The majority received annual examination of HbA1c (86%) and SBP (86%), whereas this was 67% for LDL-C and 49% for ACR. Adequate management regarding the three-step pathway was observed in 73%, 53%, 46%, 41% of patients for HbA1c, SBP, LDL-C, and ACR respectively. Quality scores reduced significantly due to the second step (OR 0.43, 0.18, 0.44, 0.74), but were not much further reduced by the third step. Timely treatment evaluation occurred in 88% for HbA1c, 87% for SBP, 83% for LDL-C, and 76% for ACR. The overall score was not significantly changed by using strict time windows.

**Conclusion:**

Quality estimates of glycemic, blood pressure and cholesterol management are substantially reduced when looking at clinical pathways as compared to estimates based on commonly used simple process measures.

## Introduction

Process of care indicators are often used to assess the quality of diabetes care [1], [2]. Most of them look at specific actions in isolation, measuring processes of care such as ‘percentages of patients with type 2 diabetes who received an HbA1c test in a year’. They do not reflect the overall pathway of risk factor management as described in clinical practice guidelines, which includes (1) a periodic test of the risk factors, (2) the initiation or adjustment of drug treatment in patients with elevated risk factor levels, and (3) the subsequent evaluation of response to this treatment [3]. Estimates of quality of diabetes care show that monitoring of risk factors may reach levels of 75–95% [4], [5], whereas treatment intensification rates in subsets of patients with elevated risk factor levels may be as low as 15–57% [6]–[11]. From these studies, it is not clear how many patients receive suboptimal risk factor management considering all steps in succession. Nor is it clear how much the estimate of quality is lowered by adding the subsequent steps. Some studies have tried to quantify the overall quality of care for risk factor management using composite scores of commonly available process and outcome indicators [12], [13], but none of them have quantified the quality of the process of care as a whole.

Looking at clinical pathways, one not only assesses whether actions were taken but whether they were taken at the right time. The timing of actions, however, is not as clearly specified in clinical guidelines for diabetes [14]–[16]. Recommendations for optimal time periods can be based on evidence and expert opinion as well as feasibility for patients and health care organizations [17], [18]. For quality assessment, there is consensus that risk factors should be monitored at least annually [19]–[22]. Regarding the initiation or intensification of treatment in patients with elevated risk factor levels, no specific time periods are indicated in the guidelines. Several professionals advocate prompt action [23]–[25], whereas others consider some delay as reasonable [26]. In research on quality of diabetes care, time periods for treatment intensification range from 14 days to 6 months [6]–[8], [27]–[29]. Other studies did not clearly specify the time periods used [9], [10], [30], [31]. Regarding the subsequent evaluation of response to treatment, guideline recommendations are inconsistent, and have not been translated to process of care assessment in the field of diabetes care [19]–[22], [32].

The aim of our study is to assess the quality of diabetes care by looking at the overall pathway of testing for elevated risk factor levels, intensification of treatment, and response to treatment evaluation, and compare this with quality as reflected by the isolated steps of risk factor management. In addition, we will evaluate the impact using different definitions of timeliness on this quality assessment, and intend to propose reasonable time periods for actions as can be derived from current clinical practice.

## Methods

We conducted a longitudinal observational study using data collected from the Groningen Initiative to Analyse Type 2 diabetes Treatment (GIANTT) database. At the time of our study, the GIANTT database consisted of anonymous longitudinal data collected from medical records of more than 20,000 patients with type 2 diabetes registered in 100 general practices in the north of the Netherlands. The database includes all general practice prescriptions, routine laboratory measurements and physical examinations as documented in the electronic patient records. Our study covers the period from the beginning of 2007 till the end of 2008. Included were patients diagnosed with type 2 diabetes before 1^st^ January 2007, who were managed for diabetes by their general practitioner, and had complete follow-up during the study period.

### Outcome measures

The outcome measures were quality of care measures derived from the prevailing guideline recommendations at the time of our study. This type of measures has been found face and content valid [21], [32], [33]. For each of the risk factors, we calculated percentages of (1) all patients with at least one risk factor test in 2007, (2) patients with an elevated risk factor in 2007, and not on maximum treatment or returning to control, who received a related treatment intensification, (3) patients with such a treatment intensification who received a subsequent evaluation of response to treatment, and finally (4) patients receiving adequate care for all three steps of this clinical pathway. Patients were considered *‘adequately managed’* when they received care as indicated by guideline recommendations [16], including also patients with adequate risk factor levels in whom no further steps need to be taken, and patients on maximum treatment.

We included the following risk factors: HbA1c, systolic blood pressure (SBP), LDL-cholesterol (LDL-C), and albumin/creatinine ratio (ACR). We used recommendations from the prevailing Dutch guidelines to define the actions that should be taken [16]. They recommend that these risk factors should be tested every year in all patients with type 2 diabetes. The first elevated test result of a risk factor in 2007 was considered as the index moment for further actions if it did not return to control within 120 days. Intensification of treatment is recommended for patients with HbA1c>7%; SBP≥140 mmHg; LDL-C>2.5 mmol/l; ACR (males)≥2.5 mg/mmol; ACR (females)≥3.5 mg/mmol [16]. Intensification of treatment was defined as the start or addition of a new drug class or a dosage increase of respectively glucose lowering, blood pressure lowering, and lipid-lowering medication. For elevated ACR levels, the start or dosage increase of a renin-angiotensin-aldosterone system intervention (RAAS-i) was defined as intensification of treatment. Evaluation of response to treatment was defined as testing of the corresponding risk factor after treatment intensification. For glucose lowering medication, either testing of HbA1c or fasting blood glucose (FBG) testing was considered as evaluation of response to treatment. Although the primary reason for this test might not be to evaluate a treatment response, the test results reflect the risk factor level after a change of treatment and we assume that this is taken into account as such by the health care provider.

Patients on maximum treatment were excluded from the total number of patients with elevated risk factor levels when calculating percentages of patients who received intensification of treatment. Maximum treatment was defined according to guideline [16]. For glucose-lowering medication, prescription of insulin was considered as having reached maximum treatment. For blood-pressure-lowering medication, prescription of 3 or more drugs from different classes at maximum maintenance dosage was considered as maximum treatment. For lipid-lowering medication, prescription of one drug at maximum dosage was considered maximum treatment. Prescribing of either an angiotensin-converting enzyme inhibitor or an angiotensin-II-receptor antagonist at maximum dosage was considered maximum treatment for elevated ACR levels. Dosage recommendations were obtained from the *Dutch Pharmacotherapy Compendium* [34].

### Time periods for quality assessment

We first set wide periods of 180 days for subsequent steps of action as have been used in previous studies [6]–[8], [28]. Next, we defined the following time periods for treatment intensification: prompt reaction (within 30 days), lenient reaction (within 31–120 days), and delayed reaction (within 121–180 days). The time period of 30 days for prompt action takes into account that some time may pass between the date of a risk factor test in the medical record and the actual prescriber-patient contact when treatment can be intensified. As lenient time period, we used a period of 120 days, as suggested in some studies. This takes delays until the next regular visit due to competing demands or clinical uncertainty into account [9], [25], [26]. For response to treatment evaluation, we set the following time periods: too early reaction which could be tests conducted for other reasons (within 42 days for HbA1c, within 14 days for SBP, and within 21 days for LDL-C), timely reaction including a first or second test after treatment intensification (43–120 days for HbA1c or 1–120 days for FBG, 15–120 days for SBP, 22–120 days for LDL-C, and within 365 days for ACR), and delayed reaction (121–180 days for HbA1c/FBG, SBP, LDL-C). The time periods for too early reactions were based on guideline recommendations [16]. Changes in HbA1c levels should be measured after a minimum period of 42 days, because HbA1c reflects average glycemia over the preceding 6 weeks. No limitations are stated for evaluation of changes in FBG level. Changes in SBP level in response to treatment should be measured after 2–4 weeks. LDL-C should be measured after several weeks, which we considered to be at least 3 weeks. Regarding evaluation of ACR in response to RAAS-i treatment there is only the recommendation for annual audit.

### Time periods derived from clinical practice

Time periods for actions can be set using evidence and expert opinion as well as feasibility for patients and health care organizations [17], [18]. To determine reasonable time periods for treatment intensification, we assessed feasibility by comparing our predetermined time periods with the actual distribution of cases observed for such intensifications. We calculated the number of patients receiving treatment intensification over time, using 10-day intervals after the index date. For response to treatment evaluation, we calculated the number of patients receiving a subsequent risk factor test over time after the treatment intensification date, and assessed whether a change in the risk factor levels could be observed. We used 10-day intervals for HbA1c and SBP and 20-day intervals for LDL-C and ACR (to gain higher numbers of eligible cases) after the date of treatment intensification. The changes in risk factor level were calculated as the mean difference between the risk factor level after and before the treatment intensification. The timing for evaluation of response to treatment was considered too early when the mean changes in risk factor level did not yet reach a significant change.

### Analysis

Descriptive analysis are presented showing percentages of patients in each step of the clinical pathway as well adequately managed for the overall clinical pathway using (1) wide time periods of 180 days, and (2) strict time periods, including prompt and lenient reactions of treatment intensification and timely reactions of response to treatment evaluation. Using odds ratios (OR) and 95% confidence intervals (CI) we compared the proportions of patients who were adequately managed in the overall three-step pathway of risk factor management (1) with those adequately managed only regarding the first step of the clinical pathway, and (2) with those adequately managed regarding the first and second step. Furthermore, we compared the proportions of patients who were adequately managed using wide or strict time periods.

For the mean changes in risk factor levels after treatment per 10-day or 20-day interval, we present 95% confidence intervals. To test for significant differences over time we used independent t-tests.

## Results

Overall, 11176 patients diagnosed with type 2 diabetes before 1^st^ of January 2007 were available for the study, of whom 9439 (84.5%) had complete follow-up until the end of 2008 and were included in our study, whereas 1737 (15.5%) died or moved to another region. Patients were at baseline on average 66 years of age with a diabetes duration of almost 6 years (Table 1).

**Table 1:** Patient characteristics at baseline.

	Number of patients with observation (%)	Mean ± standard deviation
Age (years)	9439	66±12.1
Male gender	4493 (47.6)	
Diabetes duration (years)	9439	5.6±5.6
HbA1c (%)	8144	6.8±1.0
Systolic blood pressure (mmHg)	8140	142.8±20.5
LDL-cholesterol (mmol/l)	6264	2.4±0.9
Total cholesterol (mmol/l)	6424	4.4±1.1
Albumin/creatinin ratio (mg/mmol)	4604	4.0±15.3

### Glucose management

**Figure 1:**
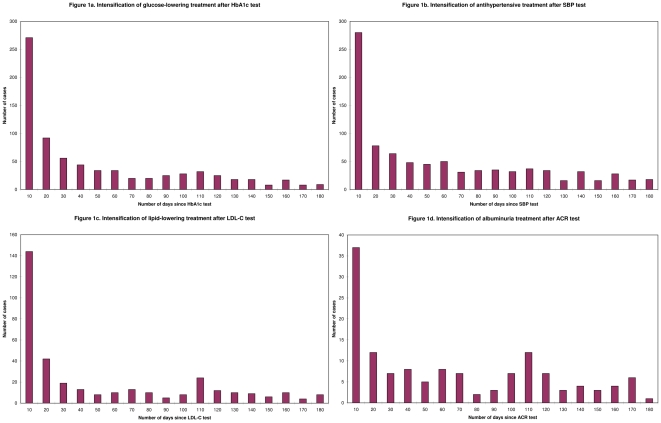
Timing of treatment intensification after risk factor test (number of patients per 10 days period).

**Figure 2:**
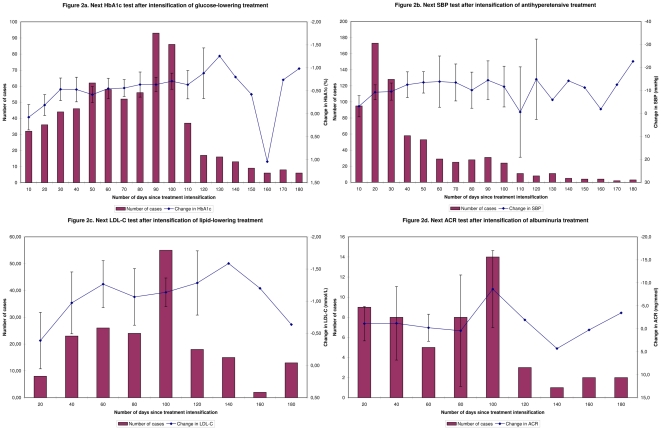
Timing of response to treatment evaluation after treatment intensification (number of patients per 10- or 20-days period in bars, and 10- or 20-day mean values of changes in risk factor levels in line graphs).

6878 (73%) patients were adequately managed in the three-step pathway using the wide time periods of 180 days (Table 2). 8144 (86%) patients received at least one HbA1c test in 2007. Of the 1975 patients above target, not returning to control and not on maximum treatment, only 759 (38%) received a treatment intensification. Treatment intensification was prompt for 419 (55%), lenient for 262 (35%) and delayed for 78 (10%) of these patients (Table 3). The highest peak for treatment intensification occurred within the first 10 days (Figure 1a). Most of patients (93%), had a next HbA1c or FBG test within 180 days after the treatment intensification (Table 2). For 563 (88%) of these patients, this was considered as a timely reaction (Table 3). Evaluation of response to treatment most often occurred close to the time of the next regular practice visit (Figure 2a). Mean changes in HbA1c significantly improved and leveled off after 20 days since treatment intensification (p = 0.04). The quality score regarding adequate management reduced significantly due to the second step but was not much further reduced by the third step (Table 2). The overall score for adequate management was not significantly higher using wide time periods (73%) in comparison to using strict time windows (72%; OR 1.06, 95% CI 0.99–1.12).

**Table 2:** Percentages of patients who received recommended care within wide time periods of 180 days.

	HbA1c	SBP	LDL-C	ACR
	n = 9439	n = 9439	n = 9439	n = 9439
Adequately managed in the clinical pathway as indicated within time periods of 180 days	6878 (73%)	4968 (53%)	4383 (46%)	3905 (41%)
*Step 1 Risk factor testing in whole population*				
Risk factor test in 2007	8144 (86%)	8140 (86%)	6264 (67%)	4604 (49%)
- elevated level	2556 (31%)	4713 (58%)	2332 (37%)	1165 (25%)
- return to control within 120 days	339 (13%)	776 (16%)	117 (5%)	187 (16%)
- maximum treatment	242 (9%)	73 (2%)	150 (6%)	227 (19%)
Odds Ratio (95%CI) of adequate management step 1 versus all 3 steps	0.43 (0.35–0.50)	0.18 (0.11–0.25)	0.44 (0.38–0.50)	0.74 (0.68–0.80)
*Step 2 Treatment intensification in patients with elevated risk factor level who are not on maximum treatment*				
	n = 1975	n = 3864	n = 2065	n = 751
Intensification of treatment within 180 days	759 (38%)	895 (23%)	355 (17%)	136 (18%)
Odds Ratio (95%CI) of adequate management of steps 1 and 2 versus all 3 steps	0.97 (0.91–1.04)	0.92 (0.86–0.97)	0.93 (0.87–0.99)	0.96 (0.91–1.02)
*Step 3 Evaluation of response to treatment in patients who received intensification of treatment*				
	n = 759	n = 895	n = 355	n = 136
Evaluation of response to treatment within 180 days	709 (93%)*	692 (77%)	184 (52%)	52 (38%)

*First test of HbA1c or fasting blood glucose observed after intensification of treatment.

**Table 3:** Percentages of patients who received care within predefined time periods.

	HbA1c	SBP	LDL-C	ACR
	n = 9439	n = 9439	n = 9439	n = 9439
Adequately managed in the clinical pathway within strict time periods*	6776 (72%)	4969 (53%)	4479 (47%)	3989 (42%)
*Step 1 Risk factor testing in whole population*				
Risk factor test in 2007	8144 (86%)	8140 (86%)	6264 (67%)	4604 (49%)
- elevated level	2556 (31%)	4713 (58%)	2332 (37%)	1165 (25%)
- return to control within 120 days	339 (13%)	776 (16%)	117 (5%)	187 (16%)
- maximum treatment	242 (9%)	73 (2%)	150 (6%)	227 (19%)
*Step 2 Treatment intensification in patients with elevated risk factor level who are not on maximum treatment*				
	n = 759	n = 895	n = 355	n = 136
- prompt reaction (within 30 days)	419 (55%)	422 (47%)	205 (58%)	56 (42%)
- lenient reaction (within 31–120 days)	262 (35%)	346 (39%)	103 (29%)	59 (43%)
- delayed reaction (within 121–180 days)	78 (10%)	127 (14%)	47 (13%)	21 (15%)
*Step 3 Evaluation of response to treatment in patients who received prompt or lenient reaction of treatment intensification*				
	n = 637	n = 589	n = 160	n = 87
- too early reaction**	39 (6%)	53 (9%)	2 (1%)	-
- timely reaction (within 120 days without too early)	563 (88%)	514 (87%)	132 (83%)	87 (76%)+
- delayed reaction (within 121–180 days)	35 (6%)	22 (4%)	26 (16%)	-

*Time periods of prompt and lenient reactions of treatment intensification and timely reaction of response to treatment evaluation.

**Within 42 days for HbA1c, no restriction for FBG, within 14 days for SBP, and within 21 days for LDL-cholesterol, no recommendations for too early ACR.

+ Within 1 year period.

### Blood pressure management

4968 (53%) patients were adequately managed in the clinical pathway using the wide time periods of 180 days (Table 2). 8140 (86%) patients received at least one SBP test in 2007. Of the 3864 patients above target, not returning to control and not on maximum treatment, only 895 (23%) received a treatment intensification. Treatment intensification was prompt for 422 (47%), lenient for 346 (39%), and delayed for 127 (14%) of these patients (Table 3). The highest peak for treatment intensification occurred within the first 10 days (Figure 1b). Most of patients (77%), had a next SBP test within 180 days after the treatment intensification (Table 2). For 514 (87%) of these patients, this was considered as timely (Table 3). Evaluation of response to treatment most often occurred within 30 days after index visit to health care provider (Figure 2b). Mean changes in SBP significantly improved and leveled off after 10 days since treatment intensification (p = 0.03). The quality score regarding adequate management reduced significantly due to the second step, and slightly by the third step (Table 2). The overall score for adequate management was not significantly higher using wide time periods (53%) in comparison to using strict time windows (53%; OR 1.00, 95% CI 0.94–1.06).

### LDL-cholesterol management

4383 (46%) patients were adequately managed in the clinical pathway using the wide time periods of 180 days (Table 2). 6264 (67%) patients received at least one LDL-C test in 2007. Of the 2065 patients above target, not returning to control and not on maximum treatment, only 355 (17%) received a treatment intensification. Treatment intensification was prompt for 205 (58%), lenient for 103 (29%), and delayed for 47 (13%) of these patients (Table 3). The highest peak for treatment intensification occurred within the first 10 days (Figure 1c). More than half of patients (52%), had a next LDL-C test within 180 days after treatment intensification (Table 2). For 132 (83%) of these patients, this was considered as timely (Table 3). Evaluation of response to treatment most often occurred close to time of the next regular practice visit (Figure 2c). Mean changes in LDL-C showed a trend to improvement and leveled off after 20 days since treatment intensification (p = 0.06). The quality score regarding adequate management reduced significantly due to the second step, and slightly by the third step (Table 2). The overall score for adequate management was not significantly different using wide time periods (46%) in comparison to using strict time windows (47%; OR 0.96, 95% CI 0.90–1.02).

### Albuminuria management

3905 (41%) patients were adequately managed in the clinical pathway using the time periods of 180 days (Table 2). 4604 (49%) patients received at least one ACR test in 2007. Of the 751 patients above target, not returning to control and not on maximum treatment, only 136 (18%) received a treatment intensification. Treatment intensification was prompt for 56 (42%), lenient for 59 (43%) and delayed for 21 (15%) of these patients (Table 3). The highest peak for treatment intensification occurred within the first 10 days (Figure 1d). 52 (38%) patients had a next ACR test within 180 days after treatment intensification (Table 2) and 87 (76%) patients had a next ACR test within a year, which was considered as timely (Table 3). Mean changes in ACR did not show clear improvement on the timeline within 100 days (p = 0.98). The quality score regarding adequate management reduced significantly due to the second step but was not much further reduced by the third step (Table 2). The overall score for adequate management was not significantly different using wide time periods (41%) in comparison to using recommended strict time windows (42%; OR 0.96, 95% CI 0.91–1.02).

## Discussion

Quality of risk factor management in diabetes looking at the three-step process of care pathway showed that up to 59% of the patients may receive less care than recommended according to the guidelines. Specifically, quality estimates of glycemic, blood pressure and cholesterol management were substantially reduced when looking at clinical pathways as compared to estimates based on commonly used simple process measures. The assessed quality was higher for glycemic management than for blood pressure or cholesterol and especially albuminuria management, regardless of the time periods used for defining the quality. Suboptimal quality seems mostly driven by lack of treatment intensification for all risk factors, and by lack of risk factor testing for cholesterol and albuminuria management. Although treatment intensifications often occurred within 30 days, taking into account actions until the next regular practice visit almost doubled the estimated quality of treatment intensification for patients with elevated risk factor levels. The percentages of patients who received the recommended care did not significantly increase when further extending time periods for quality assessment up to 180 days.

At each step of the clinical pathway patients received less care than recommended. Regarding risk factor testing, in particular fewer patients received at least one test of LDL-C and ACR within a year. Previous studies also showed room for improvement regarding quality of testing for cholesterol and albuminuria in diabetes patients [8], [35], [36]. This may be explained by the fact that routine testing of cholesterol and albuminuria is recommended once a year whereas this is half-yearly or quarterly for glycemia and blood pressure. Tests conducted once yearly have a higher chance of falling just outside a fixed observation period of 12 months. This would support the choice made in the British Quality and Outcome Framework system to use periods of 15 months instead of 12 months for quality assessment of risk factor testing [19].

Regarding treatment intensification among patients with elevated risk factors level, the low rates observed are consistent with previous studies in the Netherlands [7], [8], [11] and in other health care settings [31], [37]–[40]. Patients received more treatment intensification in response to elevated levels of HbA1c than SBP, LDL-C and ACR, which is also in line with previous studies [7], [11], [41]. Allowing for treatment intensification on the next regular visit, i.e. within 120 days in The Netherlands, covers more than 85% of the intensifications occurring after elevated levels. This could be considered as a reasonable time period based on current clinical practice [17], [18]. In general, however, the intensification rates remained low. This shows that delay in action is not the most important factor for the observed low rates. Other explanations have been suggested, such as uncertainty regarding elevated risk factor levels, disagreement with guideline recommendations, the inability to intensify treatment in some patients, and refusal by patients [9], [42], [43]. Previous studies in our study population showed, however, that factors such as medication burden and medication non-adherence were not associated with lower treatment intensification rates [11], [44]. We excluded patients who were already on maximum treatment or returned to control, but there may still be some patients who did not tolerate or wanted to receive a treatment intensification. This would result in underestimates of the quality of care.

The third step of the clinical pathway, response to treatment evaluation, has not been studied before as part of quality assessment in diabetes management. Our findings demonstrated that, similar to risk factor testing in general, response to treatment evaluation is conducted more often for HbA1c and SBP management than for LDL-C and ACR management. This evaluation is also liable to setting of different time periods. Evaluation of treatment can be conducted not only too late but also too early. Too early evaluation can satisfy the definition of a quality indicator but be irrelevant from a clinical point of view. Few patients received an HbA1c test within six weeks after intensification of glucose-lowering treatment, which is too early according to Dutch guideline [16]. Other guidelines, such as from the American Diabetes Association, consider longer periods of 2–3 months over which HbA1c reflects changes [14]. In turn, we observed improvements in mean HbA1c levels already after a period of 20 days, which could indicate that for clinical practice assessment a minimum period of 3 weeks could be adequate for response to glucose-lowering treatment evaluation. For evaluating response to antihypertensive treatment, guidelines recommend to measure the SBP after 2–4 weeks. This corresponds with improvements in mean SBP levels we observed after 10 days, indicating that a minimum period of 2 weeks could be used as adequate for response to antihypertensive treatment evaluation. For lipid-lowering treatment, the Dutch guideline states that an evaluation should take place after several weeks, which we defined as 3 weeks [16]. The American guideline recommends a minimal period of 6 weeks for response to treatment evaluation [45]. Our findings indicate that a minimum period of 3 weeks could be used to reflect adequate response to lipid-lowering treatment evaluation. Regarding evaluation of response to RAAS-i treatment in case of albuminuria, it has only been stated that repeated testing is reasonable [46], but guidelines recommend only annual routine testing of ACR [14], [16]. In our study, no firm conclusions can be drawn due to the small numbers of patients with recurrent ACR tests.

The strength of our study is that it was conducted using a non-restricted population of primary care patients with type 2 diabetes using data from medical records. It reflects quality of diabetes care in the northern part of the Netherlands which may differ from other countries. It is limited to process of care assessment, whereas quality of care can also be assessed by including (intermediate) outcome measures. This is, for example, the case in the British Quality and Outcome Framework [19]. The chosen definitions of adequate care are consistent with other international and national guidelines for type 2 diabetes [14], [15], [47]–[49]. Although one might question whether treatment intensification is needed or wanted in all patients above the defined target values, especially given recent findings of published clinical trials [50], [51], our study reflects quality of care as measured according to recommendations in prevailing diabetes guidelines at the time of our study. The quality measures we used were derived from these guideline, and as such can be considered content valid. There is, however, limited evidence for their predictive validity regarding patient outcomes [52]. We considered changes in treatment after one elevated level as adequate, since in this type of longitudinal observational study this can already be a recurrent elevated risk factor measurement.

We based our proposed time periods on a combination of guideline recommendations and feasibility in daily practice. Ultimately, definitions of the optimal time periods should be based on their impact on health outcomes. The effect of the time period definitions on quality assessment is likely to depend on reimbursement, and local or national organization and agreements for regular or standard care. In the Netherlands, as in many other countries, diabetes patients usually have a regular visit with their health care provider every three months. Our predefined time periods may be less applicable for settings where this is not the case. To assess too early response to treatment evaluation, we chose 10-day and 20-day intervals to have sufficient numbers of patients on the one hand, and clinically meaningful time intervals on the other. For albuminuria, however, this resulted in small numbers of patients per interval and unreliable outcome estimates.

Study data were obtained from electronic patient records of general practices using validated procedures [53]. Such patient records provide detailed clinical information, however, they may be incomplete and contain misclassifications. Especially, tests and drugs prescribed by specialists in the hospital can be missed. Since we included only patients who are primarily managed by their general practitioner, this will be uncommon for our study population. Furthermore, dates of tests in patient records may be imprecise, either reflecting the date when the test was performed or the date when the result was received in the practice. This was taken into account by defining prompt reaction to testing as any action within a period of 30 days.

In conclusion, looking at the overall pathway of risk factor management in diabetes significantly lowers estimates of quality as compared to the assessment based on commonly used simple process measures. Our study showed that this reduction is mostly driven by lack of treatment intensification for all risk factors. Based on our findings from clinical practice, a period of 12 months may be too short for assessing annual testing of risk factors such as cholesterol and albuminuria. For assessing intensification of treatment and response to treatment, it seems reasonable to allow for the next routine diabetes visit. Extension of the time periods for quality assessment up to half a year did not significantly influence the quality estimates.
